# Supplementary material for: A Case of a Malignant Lymphoma Patient Persistently Infected with SARS-CoV-2 for More than 6 Months
Source: Medicina (Kaunas). 2023 Jan 4;59(1):108. doi: 10.3390/medicina59010108 (PMC9864643; doi:10.3390/medicina59010108)
Supplement: Supplementary file 1 [file medicina-59-00108-s001.zip › medicina-2120670-supplementary.pdf]

Table S1. Worldwide representative SARS-CoV-2 sequences registered in GISAID (https://www.gisaid.org) from September 2020 to March 2021.

No.	Collection Date	GISAID Accession Number	Viral Name	Region	Country
1	2021-02-08	EPI_ISL_1425429	hCoV-19/Japan/PG-25371/2021	Asia	Japan
2	2020-12-26	EPI_ISL_1168867	hCoV-19/Japan/PG-17557/2020	Asia	Japan
3	2020-10-31	EPI_ISL_1431453	hCoV-19/Japan/PG-27364/2020	Asia	Japan
4	2020-12-26	EPI_ISL_2498317	hCoV-19/USA/MT-WL-787/2020	North America	Canada
5	2020-10-29	EPI_ISL_8876129	hCoV-19/USA/MT-WL-787/2020	North America	USA
6	2020-12-31	EPI_ISL_1122088	hCoV-19/USA/OR-OHSU-8861/2020	North America	USA
7	2020-12-09	|EPI_ISL_731649	hCoV-19/England/ALDP-C3AFD7/2020	Europe	United Kingdom
8	2020-09-24	|EPI_ISL_580090	hCoV-19/England/ALDP-9EAC0F/2020	Europe	United Kingdom
9	2020-12-14	EPI_ISL_1895002	hCoV-19/Denmark/DCGC-85683/2020	Europe	Denmark
10	2021-03-11	EPI_ISL_1367383	hCoV-19/USA/OH-CDC-QDX22904787/2021	North America	USA
11	2020-09-16	EPI_ISL_566175	hCoV-19/England/ALDP-9CDB05/2020	Europe	United Kingdom
12	2021-02-10	EPI_ISL_4964957	hCoV-19/USA/NY-CDC-LC0017051/2021	North America	USA
13	2020-12-30	EPI_ISL_1427139	hCoV-19/Japan/PG-22363/2020	Asia	Japan
14	2021-03-29	EPI_ISL_1597109	hCoV-19/Netherlands/OV-RIVM-24229/2021	Europe	Netherlands
15	2020-12-28	EPI_ISL_5143139	hCoV-19/United_Arab_Emirates/BTC-4593/2020	Asia	United Arab Emirates
16	2020-09-24	EPI_ISL_639971	hCoV-19/Russia/SVE-RII-MH2829S/2020	Europe	Russia
17	2020-10-16	EPI_ISL_2491729	hCoV-19/Brazil/BA-FIOCRUZ-33273/2020	South America	Brazil
18	2020-12-17	EPI_ISL_1423912	hCoV-19/Spain/CT-IBV-98016339/2020	Europe	Spain
19	2021-03-03	EPI_ISL_8593673	hCoV-19/Italy/TAA-7830034987/2021	Europe	Italy
20	2021-02-01	EPI_ISL_1091227	hCoV-19/Croatia/HRV000_30/2021	Europe	Croatia
21	2020-11-06	EPI_ISL_1202956	hCoV-19/USA/TX-HMH-MCoV-17245/2020	North America	USA
22	2021-01-04	EPI_ISL_2598056	hCoV-19/USA/MD-MDH-2614/2021	North America	USA
23	2021-02-22	EPI_ISL_1272738	hCoV-19/USA/WA-CDC-2-3980593/2021	North America	USA
24	2021-03-08	EPI_ISL_1591270	hCoV-19/Croatia/1227/2021	Europe	Croatia
25	2021-03-27	EPI_ISL_2535773	hCoV-19/Malaysia/UNIMAS-16855/2021	Asia	Malaysia
26	2021-03-29	EPI_ISL_1440831	hCoV-19/Germany/NW-RKI-I-057128/2021	Europe	Germany
27	2021-03-15	EPI_ISL_1755001	hCoV-19/France/IDF-HMN-21032230485/2021	Europe	France

Table S2. Representative SARS-CoV-2 sequence from Fukuoka Prefecture, Japan registrated in GISAID(https://www.gisaid.org) from September 2020 to March 2021.

No.	GISAID Accession Number	Viral Name	Collection Date
1	EPI_ISL_900775	hCoV-19/Japan/PG-10038/2020	2020-09-30
2	EPI_ISL_897507	hCoV-19/Japan/PG-13193/2020	2020-11-25
3	EPI_ISL_894680	hCoV-19/Japan/PG-15874/2020	2020-11-25
4	EPI_ISL_894720	hCoV-19/Japan/PG-15916/2020	2020-12-16
5	EPI_ISL_894746	hCoV-19/Japan/PG-15942/2020	2020-12-23
6	EPI_ISL_894748	hCoV-19/Japan/PG-15944/2020	2020-12-23
7	EPI_ISL_1128441	hCoV-19/Japan/PG-16439/2020	2020-12-23
8	EPI_ISL_1128926	hCoV-19/Japan/PG-16882/2020	2020-12-30
9	EPI_ISL_1319755	hCoV-19/Japan/PG-19044/2021	2021-01-06
10	EPI_ISL_1125030	hCoV-19/Japan/PG-20425/2021	2021-01-06
11	EPI_ISL_1125032	hCoV-19/Japan/PG-20426/2021	2021-01-06
12	EPI_ISL_1426746	hCoV-19/Japan/PG-21729/2021	2021-01-06
13	EPI_ISL_1426795	hCoV-19/Japan/PG-21781/2021	2021-01-13
14	EPI_ISL_1472132	hCoV-19/Japan/PG-22093/2021	2021-01-13
15	EPI_ISL_1430155	hCoV-19/Japan/PG-24686/2021	2021-01-27
16	EPI_ISL_2014929	hCoV-19/Japan/PG-27489/2021	2021-02-10
17	EPI_ISL_1472251	hCoV-19/Japan/PG-29145/2021	2021-01-13
18	EPI_ISL_2014974	hCoV-19/Japan/PG-29814/2021	2021-03-10
19	EPI_ISL_2014999	hCoV-19/Japan/PG-30022/2021	2021-03-03
20	EPI_ISL_2015005	hCoV-19/Japan/PG-30028/2021	2021-03-03
21	EPI_ISL_1930039	hCoV-19/Japan/PG-30179/2021	2021-03-03
22	EPI_ISL_2015027	hCoV-19/Japan/PG-31600/2021	2021-03-03
23	EPI_ISL_2015052	hCoV-19/Japan/PG-32597/2021	2021-03-10
24	EPI_ISL_3191930	hCoV-19/Japan/PG-68292/2021	2021-01-13
25	EPI_ISL_3191997	hCoV-19/Japan/PG-68524/2021	2021-01-13
26	EPI_ISL_3199801	hCoV-19/Japan/PG-71414/2021	2021-03-03
27	EPI_ISL_3874861	hCoV-19/Japan/PG-74222/2021	2021-01-13
28	EPI_ISL_687534*	hCoV-19/Japan/PG-8435/2020	2020-09-11
29	EPI_ISL_687551*	hCoV-19/Japan/PG-8452/2020	2020-09-09
30	EPI_ISL_687540*	hCoV-19/Japan/PG-8441/2020	2020-09-03

*Sequence of hospital infection clusters.
